# Assessing rice production sustainability performance indicators and their gaps in twelve sub-Saharan African countries

**DOI:** 10.1016/j.fcr.2021.108263

**Published:** 2021-09-15

**Authors:** Aminou Arouna, Krishna Prasad Devkota, Wilfried Gnipabo Yergo, Kazuki Saito, Benedicta Nsiah Frimpong, Patrice Ygue Adegbola, Meougbe Ernest Depieu, Dorothy Malaa Kenyi, Germaine Ibro, Amadou Abdoulaye Fall, Sani Usman

**Affiliations:** a Africa Rice Center (AfricaRice), 01 BP 2551, Bouaké, Cote d’Ivoire; b Council for Scientific and Industrial Research - Crops Research Institute (CSIR-CRI), Kumasi, Ghana; c Institut National des Recherches Agricoles du Bénin (INRAB), Cotonou, Benin; d Centre National de Recherche Agronomique (CNRA), Gagnoa, Cote d’Ivoire; e Institut de Recherche Agricole pour le Développement (IRAD), Yaoundé, Cameroon; f Institut National de la Recherche Agronomique du Niger (INRAN), Niamey, Niger; g Institut Sénégalais de Recherches Agricoles (ISRA), Saint-Louis, Senegal; h National Agricultural Extension and Research Liaison Services (NAERLS), Ahmadu Bello University, Zaria, Nigeria

**Keywords:** Yield gap, Net profit, Nitrogen use efficiency, Phosphorus use efficiency, Irrigated lowland, Rainfed lowland

## Abstract

The benchmarking and monitoring of rice production performance indicators are essential for improving rice production self-sufficiency, increasing profitability, reducing labor requirements, optimizing fertilizer inputs, engaging youths in rice production, and increasing the overall sustainability of smallholder rice production systems in countries in sub-Saharan Africa (SSA). In this paper, we quantified five sustainability performance indicators (grain yield, net profit, labor productivity, and nitrogen (N) and phosphorus (P) use efficiencies) to benchmark rice production systems in SSA. Data were collected between 2013–2014 from 2907 farmers from two rice production systems (irrigated and rainfed lowlands) across five agroecological zones (arid, semiarid, humid, subhumid and highlands) in 12 countries (Benin, Cameroon, Cote d’Ivoire, Ghana, Madagascar, Mali, Niger, Nigeria, Senegal, Sierra Leone, Tanzania and Togo). The exploitable gap for each indicator (the difference between the mean of 10 % highest-yielding farms and the mean-yielding farms) was calculated across the countries, the two production systems and agroecological zones. The mean yield varied widely between 2.5 to 5.6 t ha^−1^ and 0.6 to 2.3 t ha^−1^ in irrigated and rainfed lowlands, respectively, with an average yield of 4.1 and 1.4 t ha^−1^, respectively. Across the country-production system combinations, there were yield gaps of 29–69 %, profit gaps of 10–89 %, and labor productivity gaps reaching 71 %. Yield, profit, and labor productivity were positively correlated. They were also positively correlated with N and P fertilizer application rate, but not with N and P use efficiencies. Only between 34–44 % of farmers had desirable ranges in N- or P-use efficiencies in the two production systems. All sites for rainfed lowlands were characterized by low-yield and large gaps in yield, profit, and labor productivity, whereas irrigated lowlands in some countries (Madagascar, Mali, and Togo) have similar characteristics as rainfed ones. We conclude that there is an urgent need to disseminate precision nutrient management practices for optimizing nutrient use efficiency and enhancing rice performance indicators especially in rainfed lowlands as well as low-yielding irrigated lowlands. Furthermore, we propose recommendations for specific categories (i.e. farmer, rice production system, agroecological zone and country) to close performance indicator gaps and to allow the production at scale to achieve rice self-sufficiency in SSA.

## Highlights

• Five indicators for rice production and their gaps were assessed in 12 countries.
• The indicators include yield, profit, labor productivity, N- and P-use efficiencies.
• Mean yield varied: 2.5–5.6 t ha^−1^ in irrigated and 0.6–2.3 t ha^−1^ in rainfed.
• There were yield gaps of 29–69 % and profit gaps of 10–89 %.
• Less than 50 % of farmers had desirable ranges in N- or P-use efficiencies.

## 1 Introduction

Rice is one of the most important basic crops in sub-Saharan Africa (SSA). The rice sector is considered as an engine for economic growth in SSA, as it has the potential to contribute to creating wealth and jobs, ensuring food security, reducing economic migration from Africa, and ensuring social stability (Seck et al., 2012). However, these potential benefits remain unrealized despite the existence of national objectives in SSA countries targeted at achieving rice self-sufficiency.

Many countries in SSA have made significant efforts to increase domestic rice productivity and production by encouraging the adoption of new and improved varieties and good agricultural practices. Consequently, 71 % increase in paddy rice production during 2007–2012 was attributed to yield increases, and 29 % was attributed to harvested-area expansion (Saito et al., 2015). However, between 2012 and 2018, the increase in yield was only 1.19 % annually, compared to an annual increase of 1.47 % between 2007 and 2012 (Arouna et al., 2021a). Despite the various policies implemented to boost local production, especially after the 2007/2008 global food crisis, local production in SSA has not been sufficient to meet the increasing demands of the population.

Although significant efforts and investments had been made in rice research and development over the past 50 years, rice production in SSA is still characterized by low productivity. The average yield in 2018 in the region was approximately 2.28 t ha^−1^, compared to the average of 4.61 t ha^−1^ in Asia (USDA, 2020). The region has an exploitable yield gap of 2−10 t ha^-1^ (Dossou-Yovo et al., 2020). In SSA, the low yield constitutes one of the main challenges of rice production and is attributed to several factors. Among these, poor agricultural practices including land preparation, seed, crop establishment, nutrient management, and weed management limit on-farm yield (Dossou-Yovo et al., 2020; Niang et al., 2017; Saito et al., 2015; Tanaka et al., 2015, 2017). In addition, abiotic, biotic and socioeconomic constraints have been frequently reported to reduce rice yield in both irrigated and rainfed production environments (Diagne et al., 2013), especially with the increasing negative effect of climate change (van Oort and Zwart, 2017). However, apart from grain yield (Niang et al., 2017; Tanaka et al., 2017), little information is available for the benchmarking of performance indicators (PIs), e.g., the labor productivity, nitrogen use efficiency (NUE) and phosphorus use efficiency (PUE), of rice production systems in SSA. These PIs are among those defined by the Sustainable Rice Platform (SRP) for sustainability of rice production (SRP, 2015). These PIs are also included in a framework of agronomic gain key PIs, which was recently proposed by Excellence in Agronomy 2030 Initiative (Saito et al., 2021). Sustainable rice production requires the improvement and optimization of these PIs without major trade-off among them (Devkota et al., 2019, 2020; SRP, 2015). In SSA countries, maintaining and improving the sustainability of smallholder rice production is important for achieving Sustainable Development Goal (SDG), i.e. end poverty (SDG #1), end hunger, achieve food security and improve nutrition, and promote sustainable agriculture (SDG #2), gender equality (SDG #5), ensure sustainable consumption and production pattern (SDG #12), take urgent action to combat climate change and its impact (SDG #13) (UNDP, 2017). Quantifying the PIs of rice production is important for closing large yield and profit gaps through optimal resource use. Additionally, assessment of such PIs is required in order to establish intervention priorities (baseline/benchmarking and target), provide specific recommendations and practical guidelines to drive improvements in crop production systems and monitor progress due to agronomic interventions or policy supports over time and location. Thus, the objective of this paper is to assess the PIs for sustainable rice production across countries, production systems and agroecological zones (AEZ) in SSA.

For brevity, aligning with this special issue, this paper focuses on five farm-level economic and environmental indicators that rice agronomic interventions significantly addressed, namely, grain yield, net profit, labor productivity, NUE and PUE in SSA as a case study. The contribution of this paper to the literature is twofold. First, the quantification and comparison of rice production indicators are essential for improving the sustainability of smallholder rice production systems in SSA. Although such quantitative assessments for other parts of the world have been published in the literature (Devkota et al., 2019), studies that quantify rice production PIs in SSA are scarce. Literature exists on assessment of grain yield across rice growing environments in SSA (Tanaka et al., 2017; Niang et al., 2017; Senthilkumar et al., 2020), labor productivity and NUE in farmers’ fields in a few sites (Paresys et al., 2018; Wopereis et al., 1999). However, studies assessing trade-off among indicators (labor productivity, net profit, NUE and PUE) and with the aim of making rice production system sustainable at major rice production countries and AEZ in SSA are lacking. Second, our assessment is more holistic with wider geographical coverage (12 countries), two production systems based on water conditions and management practices (irrigated and rainfed lowlands), five AEZs, and three farmer categories based on yield performance. Unlike the existing literature, which is focused mainly on irrigated and intensively managed rice production systems (Devkota et al., 2020), this study compares rice production in both irrigation and rainfed lowland systems with sub-optimal crop and input management practices. These two perspectives lead to policy and management recommendations as well as suggestions for future research directions to improve the sustainability of rice production and achieve rice self-sufficiency in SSA.

## 2 Materials and methods

### 2.1 Survey design and sampling

**Fig. 1:**
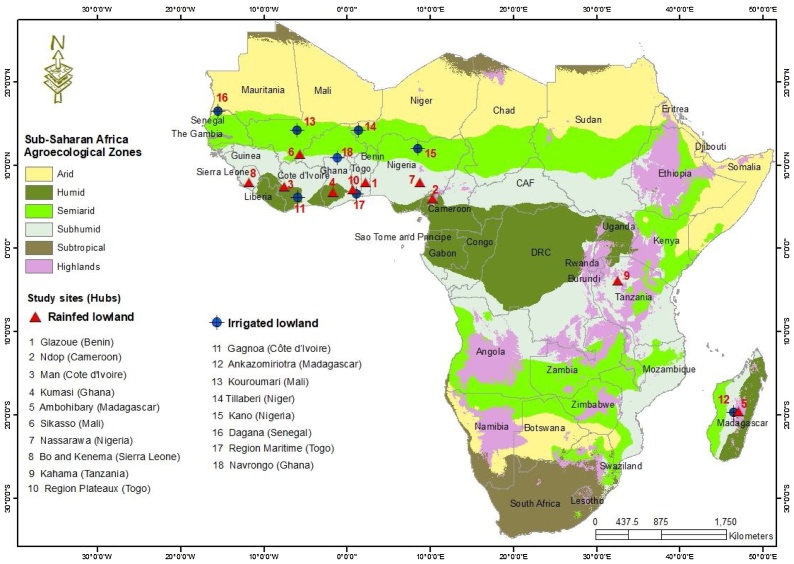
Surveyed areas in sub-Saharan Africa.

This study used data collected by Africa Rice Center (AfricaRice) for the 2013–2014 growing season from rice sector development hubs in 12 countries in SSA (Benin, Cameroon, Cote d’Ivoire, Ghana, Madagascar, Mali, Niger, Nigeria, Senegal, Sierra Leone, Tanzania and Togo) (Fig. 1). Rice sector development hubs are the main rice production areas where rice research innovations are integrated across the rice value chain to achieve development outcomes and impacts (Zossou et al., 2017). The number of rice sector development hubs per country was selected by National Agricultural Research System (NARS) partners based on the main production system and the quantity of rice produced. The survey for this study was conducted in one or two hubs (hereinafter referring as site) in each country depending on the production systems (irrigated lowland; IL and rainfed lowland; RL). In six countries (Cote d’Ivoire, Ghana, Madagascar, Mali, Nigeria, and Togo), data were collected from two hubs (one IL production system and one RL production system). Data were collected from one hub in the other six countries, i.e., two countries (Niger and Senegal) with IL and four countries (Benin, Cameroon, Sierra Leone, and Tanzania) with RL. Therefore, data were collected at 18 site–production system combinations (the IL in 8 countries and the RL in 10 countries). The 18 rice production systems were in 5 AEZ (arid, semiarid, humid, subhumid and highlands) (Fig. 1).

A multistage random sampling technique was used to select farmers to interview. In the first stage, a list of all villages that produce rice using the main production system of each hub was obtained. In each hub, the number of villages sampled was proportional to the total number of villages, and the sampled villages were randomly selected. A list of all rice farmers in each selected village was collected with the help of extension agents and national partners. Ten farmers were randomly selected from each village. In total, data from 2907 rice farmers in 12 countries were included in the analysis. Of the 2907 farmers analyzed in this paper, 1011 cultivated rice in IL, and 1896 cultivated rice in RL production system (Table 1).

**Table 1:** Characterization of rice production inputs in irrigated lowland production systems and in irrigated versus rainfed lowland production systems in SSA countries.

	Overall		Irrigated							
	Irrigated lowland	Rainfed lowland	Cote d’Ivoire (Gagnoa)	Ghana (Navrongo)	Madagascar (Ankazomiriotra)	Mali (Kouroumari)	Niger (Tillaberi)	Nigeria (Kano)	Senegal (Dagana)	Togo (Maritime)
Household rice area (ha)	1.24^a^	2.25^b^	0.63^ab^	1.15^abc^	0.27^a^	3.90^e^	1.59^bcd^	3.00^de^	1.24^b^	2.21^cd^
	(3.21)	(3.65)	(0.31)	(0.93)	(0.88)	(2.64)	(5.02)	(2.40)	(1.88)	(2.43)
Labor (Labor day ha^−1^)	90.06^a^	89.24^a^	118.76^d^	81.97^bc^	140.30^e^	36.66^a^	45.32^b^	44.58^a^	60.16^a^	116.93^c^
	(59.98)	(56.64)	(41.40)	(59.29)	(56.04)	(12.56)	(23.41)	(29.51)	(42.59)	(41.76)
Seeding rate (kg ha^−1^)	83.80^a^	71.47^b^	92.21^b^	139.09^c^	76.26^a^	71.30^a^	64.25^d^	53.08^e^	134.27^c^	83.39^b^
	(32.31)	(30.18)	(30.40)	(23.91)	(24.67)	(14.53)	(13.23)	(3.30)	(16.42)	(17.20)
Elemental N (kg ha^−1^)	40.03^a^	18.62^b^	74.62^ef^	15.30^abc^	--	20.73^bc^	84.52^f^	2.79^ab^	54.03^de^	38.10^cd^
	(68.87)	(59.67)	(141.55)	(65.80)	--	(27.11)	(86.85)	(2.68)	(55.32)	(38.53)
Elemental P (kg ha^−1^)	4.73^a^	2.32^b^	8.57^d^	2.27^abc^	--	2.25^bc^	11.98^e^	0.05^ab^	2.64^bc^	4.19^c^
	(8.64)	(7.89)	(15.48)	(7.15)	--	(2.92)	(10.77)	(0.23)	(5.61)	(4.87)
Elemental K (kg ha^−1^)	8.98^a^	4.31^b^	16.19^d^	4.12^abc^	--	4.21^bc^	22.77^e^	0.26^ab^	5.04^bc^	7.81^c^
	(16.49)	(13.61)	(29.83)	(13.66)	--	(5.47)	(20.56)	(0.45)	(10.77)	(9.32)
Certified seeds (%)	25.32^a^	25.47^a^	76.19^d^	72.73^d^	0.85^a^	8.93^ab^	52.41^e^	26.32^bc^	9.49^b^	40.32^c^
Rice transplanting (%)	90.50^a^	85.75^b^	85.71^abc^	96.97^cd^	100.00^d^	92.86^bcd^	86.82^b^	73.68^a^	76.58^a^	91.94^bc^
Mechanical Weeders (%)	48.47^a^	26.10^b^	9.52^ab^	15.15^ab^	100.00^c^	82.14^d^	14.47^b^	57.89^e^	7.59^a^	29.03^f^
Herbicide (%)	39.96^a^	39.04^a^	66.67^abc^	66.67^ab^	0.00^e^	23.21^f^	63.02^b^	89.47^cd^	53.80^a^	91.94^d^
Insecticide (%)	22.75^a^	12.63^b^	14.29^ab^	30.30^b^	23.65^b^	5.36^a^	24.76^b^	84.21^c^	1.27^a^	58.06^d^
Dikes and bunds (%)	30.86^a^	36.06^b^	47.62^ab^	60.61^bc^	--	92.86^d^	39.23^a^	78.95^cd^	32.28^a^	67.74^c^
Number of equipment types (#)	4.64^a^	3.79^b^	3.38^ab^	6.21^c^	2.49^a^	7.64^d^	4.72^b^	6.95^cd^	6.76^cd^	7.23^cd^
	(3.55)	(3.08)	(3.88)	(5.41)	(0.63)	(2.65)	(3.61)	(4.97)	(3.97)	(2.87)
Total production costs ($ ha^−1^)	270.00^a^	216.54^b^	243.50^a^	335.31^b^	108.48^d^	275.78^a^	417.19^e^	247.61^a^	382.45^c^	356.02^bc^
	(176.42)	(385.32)	(75.08)	(119.22)	(25.22)	(56.97	(181.88)	(142.73)	(89.80)	(89.71)
Average cost per kg of paddy rice ($ kg^−1^)	0.27^a^	0.33^b^	0.36^b^	0.37^b^	0.17^c^	0.31^a^	0.30^a^	0.58^d^	0.22^e^	0.54^f^
	(0.13)	(0.15)	(0.17)	(0.12)	(0.04)	(0.00)	(0.07)	(0.22)	(0.01)	(0.13)
% of farmers that do not apply N	44.41^a^	61.12^b^	23.81^abc^	27.27^a^	--	14.29^bcd^	9.97^d^	21.05^abcd^	21.52^ab^	11.29^cd^
% of farmers that do not apply P	54.70^a^	73.59^b^	23.81^cd^	42.42^b^	--	30.36^bc^	13.50^d^	94.74^a^	57.59^e^	24.19^c^
% of farmers that do not apply K	53.31^a^	69.26^b^	23.81^abc^	39.39^a^	--	28.57^ab^	12.22^c^	73.68^d^	56.33^e^	20.97^bc^
Sample size (#)	1011	1896	21	33	351	56	311	19	158	62
Hub area (km^2^)	207,734	273,270	17,580	8842	4500	23,063	89,623	43,285	19,241	6100

The same letter indicates no significant difference at the 5% level. Values in parenthesis are standard deviations. The dominant cropping system in IL is rice-rice.

### 2.2 Data collection and processing

Data collection was performed using tablets, and the data were sent via a web-based application to a central database managed by AfricaRice that allows online access to NARS partners. As data validation rules were imposed, the tablet-based data collection avoided many biases associated with paper-based questionnaires, such as mistakes in recording answers, changed values of variables, mistakes in recoding text answers for numerical variables, etc. Data were collected using a structured and pre-tested questionnaire. The household data collected included socioeconomic and demographic characteristics and information necessary to estimate the net profit, labor productivity, grain yield, NUE and PUE of the farms. Farmers were asked to recall information about field size per household, the quantity and price of all inputs (seed, fertilizer, insecticides, labor, equipment, etc.) and output (paddy rice) from the previous rainy season. The data were collected by enumerators who were trained and supervised by NARS partners and AfricaRice staff.

### 2.3 Computation of sustainability indicators and data analysis

Out of the 12 SRP indicators, the five PIs related to farm-level rice production were considered and included, viz., grain yield, net profit, labor productivity, NUE, and PUE (SRP, 2015). The net profit, labor productivity and grain yield were calculated for all 12 countries, and the NUE and PUE were calculated for 6 countries (i.e., Cote d’Ivoire, Mali, Togo, Niger, Senegal and Benin) because N and P fertilizer were not applied or were applied only at very low levels in the other countries. All the PIs were computed for the two rice production systems (IL and RL) and across five AEZ (arid, semiarid, humid, subhumid and highlands). The net profits were calculated by considering total costs, including inputs (seed, fertilizers, herbicides, irrigation water, and pesticides), machinery rental, equipment, land rental (if any), labor (family and hired) for seedling preparation, seeding, land preparation, crop establishment, weed management, fertilizer application, irrigation, harvesting, threshing, cleaning, and drying operations. The gross income was computed based on the grain yield and market price. To reduce the effect of price variability on the net profit, average price per hub was used. Then, the net profit was derived by deducting total costs from the gross income. Grain yield was estimated by dividing the total rice production by the rice area and expressed as tons per hectare (t ha^−1^). Labor productivity was computed by dividing the grain yield by the total number of labor days required for one hectare of rice production and was expressed as kg grain labor day^−1^. To calculate the PUE, the total P_2_O_5_ content of the fertilizers was multiplied by a factor of 0.4364 to convert it into the amount of elemental P (Devkota et al., 2019). The total harvested grain yield was divided by the elemental N or P value, and the NUE and PUE were expressed in terms of kg grain kg^−1^ elemental N or P. Analysis of variance (ANOVA) and t-tests were used to test the differences in the PIs among countries and production systems, respectively.

To estimate the yield gaps, farmers were categorized into three classes based on grain yield: the top decile (top 10 %), middle (middle 80 %) and bottom decile (bottom 10 %). Following Stuart et al. (2016), the exploitable yield gap was computed as the difference between the grain yield of the top decile and the mean grain yield of all farmers, and the yield gap percentage was estimated by dividing this difference by the yield of the top decile. Using the same percentile categories of the yield, the values of the gaps for the other PIs (net profit, labor productivity, NUE and PUE) were calculated. The values of the PIs are displayed as frequency distributions indicating the top, middle and bottom percentile values. A correlation analysis was conducted to assess the relationships between the five PIs and inputs use. For NUE and PUE, the optimal values were customized for the SSA countries by modifying the values as proposed by Devkota et al. (2019) for East Asia, Dobermann and Fairhurst (2000) for rice production, and EU Nitrogen Expert Panel (2015) for improving NUE. Accordingly, the range 30–100 kg grain kg^−1^ elemental N was set as optimal values for NUE, NUE < 30 as high elemental N application (wasteful), and > 100 low application (mining soil nutrients); similarly 100–400 kg grain kg^−1^ elemental P as optimal range for PUE, PUE < 100 as wasteful application, and > 400 as low (mining soil nutrients). The upper and lower boundary values for desirable NUE (100 and 30) and PUE (400 and 100) proposed for 12 African countries are similar to those for East Asian countries (100 and 30 NUE, and 350 and 100 PUE) (Devkota et al., 2019, 2021). It is noted that the method used here for computation of NUE and PUE does not account for the indigenous soil N and P supplies, nutrient from mineralization, organic fertilizers use, and fertilizers from irrigation water. Further, nitrogen inputs from biological nitrogen fixation (Ladha and Reddy, 2003) has not been accounted for. It is known that due to anaerobic conditions in flooded paddy fields, indigenous soil N and P supplies were maintained even without the use of fertilizers in long-term trials in the Philippines (Chivenge et al., 2020; Dobermann et al., 2000; Ishii et al., 2011). We recognize that assessment of indigenous soil N and P supplies is essential for generating site-specific nutrient management practices. However, this assessment and calculation of nutrient use efficiencies using indigenous soil N and P supplies are beyond the scope of this study.

To establish intervention priorities, normalized spider diagrams were created to indicate the trade-offs among the five PIs and the inputs used in both production systems and the three yield gap categories (bottom 10 %, middle 80 % and top 10 %) in each country. These tradeoffs were compared to make country-specific recommendations for high-priority interventions to close the gaps in yield, net profit, labor productivity, NUE and PUE.

## 3 Results

### 3.1 Characteristics of rice production inputs

The average rice area per household was significantly smaller in IL production system (1.24 ha) than in RL production system (2.25 ha) (Table 1). Although the percentage of farmers using certified seeds (25 %) was the same in both production systems, the quantity of seeds used was higher in IL (84 kg ha^−1^) than in RL (71 kg ha^−1^). Only 40 % of farmers in both production systems used herbicides and 26 % and 48 % used mechanical weeding (e.g. rotary weeder) in RL and IL, respectively. An average of 90 labor days ha^-1^ was used in rice production in the two production systems. On average, 61 % of farmers in RL and 44 % in IL do not apply N or apply <2 kg N ha^−1^ to rice, while up to 74 % of farmers in RL and 55 % in IL do not apply P or apply <1 kg P ha^−1^ to rice. However, higher quantities of N, P and K fertilizers were used in the IL than in the RL. This explains why rice production cost was higher in the IL ($270 ha^-1^) than in the RL ($217 ha^-1^). However, due to the higher yields from the IL, the unit cost of paddy production in the IL ($0.27 kg^-1^) is lower than that in the RL ($0.33 kg^-1^).

Across the eight countries in IL, the average rice area per household ranged from 0.27 ha (Ankazomiriotra, in Madagascar) to 3.90 ha (Kouroumari, in Mali). The lowest percentage of farmers using certified seeds was found in Madagascar (1%), followed by Mali (9%), and the highest percentages were in Cote d’Ivoire (76 %) and Ghana (73 %). The average labor use was highest in Madagascar (140 labor days ha^−1^) and the lowest in Mali (37 labor days ha^−1^). Rice production cost was the highest in Niger ($417 ha^−1^) and Senegal ($382 ha^−1^) and the lowest in Madagascar ($108 ha^−1^). Farmers in Niger applied the highest amounts of N fertilizer (80−105 kg N ha^-1^), and those in Ghana and Nigeria applied the least (<20 kg N ha^-1^) (Table 1). The low use of fertilizers in rice production in the eight countries using the IL system was also confirmed by the high percentage of farmers who did not apply N and P fertilizers or applied marginal quantities (< 2 kg N ha^-1^ and 1 kg P ha^-1^). Overall, 95 % of farmers in Nigeria (the highest percentage), 58 % in Senegal, 30 % in Mali, 24 % in Togo, 23 % in Cote d’Ivoire, and 10 % in Niger (the lowest percentage) did not apply at least one of the fertilizers (Table 1).

In RL, the average rice area per household ranged from 0.38 ha (Ambohibary, in Madagascar) and 0.61 ha (Region des Plateaux, in Togo) to 3.68 ha (Nassarawa, in Nigeria) and 8.50 ha (Kahama, in Tanzania) (Table 2). The lowest percentage of farmers using certified seeds was found in Tanzania (2%) and the highest were in Cote d’Ivoire (59 %) and Benin (57 %). The mean labor quantity used in rice production in RL ranged from the highest in Benin (134 labor day ha^−1^) to the lowest in Sierra Leone (30 labor day ha^−1^). Rice production was the most expensive in Cameroon ($386 ha^−1^) and Togo ($344 ha^−1^). Farmers in Togo and Benin applied the highest quantity of N fertilizer (80−105 kg N ha^-1^), and those in Tanzania, Nigeria and Cote d’Ivoire applied the least (<20 kg N ha^-1^) (Table 2). In the 10 countries with RL, a large percentage of farmers did not apply N and P fertilizers or applied only marginal quantities (< 2 kg N ha^-1^ and 1 kg P ha^-1^). Overall, 99 % of farmers in Tanzania and 96 % in Sierra Leone did not apply N fertilizer and 87 % in Ghana did not apply P fertilizer (Table 2).

**Table 2:** Characterization of rice production inputs in rainfed lowland production systems in SSA countries.

	Benin (Glazoue)	Cameroon (Ndop)	Cote d'Ivoire (Man)	Ghana (Kumasi)	Madagascar (Ambohibary)	Mali (Sikasso)	Nigeria (Nassarawa)	Sierra Leone (Bo &Kenema)	Tanzania (Kahama)	Togo (Plateaux)
Rice area (ha)	1.03^c^	0.65^ab^	1.05^c^	1.74^f^	0.38^a^	2.91^d^	3.68^e^	0.74^bc^	3.40^de^	0.61^abc^
	(1.87)	(0.70)	(0.96)	(1.61)	(0.97)	(2.81)	(2.86)	(0.52)	(0.11)	(0.67)
Labor (Labor day ha^−1^)	134.27^e^	122.60^d^	109.28^c^	80.35^f^	132.30^e^	34.20^ab^	45.35^b^	27.99^g^	92.15^a^	45.32^cd^
	(38.44)	(46.77)	(27.76)	(44.32)	(61.73)	(17.10)	(35.92)	(6.52)	(45.34)	(23.41)
Seeding rate (kg ha^−1^)	73.74^b^	46.51^d^	79.76^c^	130.49^e^	76.01^b^	65.11^a^	54.36^f^	62.19^a^	38.00^g^	80.79^c^
	(23.51)	(4.76)	(6.78)	(13.63)	(26.75)	(14.17)	(12.49)	(11.60)	(0.02)	(8.67)
Elemental N (kg ha^−1^)	83.33^d^	--	15.43^bc^	1.02^a^	--	27.88^c^	6.33^ab^	0.45^a^	0.01^a^	138.86^e^
	(114.01)	--	(20.42)	(1.79)	--	(29.76)	(13.82)	(2.54)	(0.19)	(139.88)
Elemental P (kg ha^−1^)	12.25^c^	--	1.61^a^	0.14^a^	--	2.24^ab^	0.24^a^	0.08^a^	--	14.10^d^
	(16.37)	--	(2.39)	(0.37)	--	(3.23)	(0.82)	(0.42)	--	(14.17)
Elemental K (kg ha^−1^)	22.13^c^	--	3.12^b^	0.41^a^	--	4.28^ab^	0.58^a^	0.14^a^	0.00^a^	26.93^d^
	(26.56)	--	(4.39)	(0.68)	--	(6.13)	(1.51)	(0.81)	(0.06)	(27.02)
Certified seeds (%)	57.26^d^	3.62^a^	59.07^d^	24.77^b^	2.73^a^	4.00^a^	27.23^bc^	32.14^c^	1.61^a^	22.41^bc^
Rice transplanting (%)	91.94^a^	25.36^d^	89.77^a^	91.44^a^	97.61^bc^	92.00^abc^	--	78.13e	89.56^a^	91.38^ab^
Mechanical weeders (%)	20.16^c^	0.00^a^	8.84^b^	25.68^d^	99.66^e^	60.00^f^	26.34^d^	0.45^a^	0.00^a^	12.07^bc^
Herbicide (%)	67.74^b^	4.35^a^	74.42^c^	77.93^cd^	0.34^a^	32.00^e^	83.48^d^	0.00^a^	0.00^a^	72.41^bc^
Insecticide (%)	0.00^a^	2.90^a^	5.12^a^	27.93^c^	12.97^b^	0.00^a^	52.23^d^	0.00^a^	0.00^a^	15.52^b^
Dikes and bunds (%)	66.13^d^	8.70^e^	29.30^a^	37.39^b^	--	32.00^abc^	47.32^c^	23.66^a^	67.07^d^	60.34^d^
Number of equipment types (#)	4.85^d^	1.07^f^	3.17^b^	7.26^e^	2.29^a^	6.68^e^	4.45^cd^	4.10^c^	2.71^ab^	3.10^b^
	(2.94)	(2.07)	(3.22)	(4.27)	(0.56)	(3.42)	(2.78)	(1.32)	(1.92)	(1.71)
Total cost of production ($ ha^−1^)	318.68^de^	385.90^e^	200.36^c^	315.92^de^	101.51^a^	262.02^bcde^	263.81^cd^	116.30^ab^	122.11^ab^	344.44^de^
	(97.01)	(1444.58)	(61.94)	(88.79)	(24.29)	(68.49)	(84.50)	(260.38)	(47.66)	(105.07)
Average cost per kg of paddy rice ($ kg^−1^)	0.29^a^	0.53^e^	0.38^d^	0.43^f^	0.17^g^	0.31^ab^	0.37^d^	0.34^bc^	0.28^a^	0.37^cd^
	(0.08)	(0.21)	(0.17)	(0.12)	(0.03)	(0.00)	(0.09)	(0.10)	(0.11)	(0.13)
% of farmers that do not apply N	14.92^c^	--	23.72^b^	54.05^d^	--	16.00^bc^	27.23^b^	95.09^a^	99.60^a^	10.34^c^
% of farmers that do not apply P	17.74^d^	--	29.30^c^	86.94^b^	--	40.00^c^	86.61^b^	96.43^a^	--	10.34^d^
% of farmers that do not apply K	16.94^d^	--	29.30^c^	68.47^b^	--	40.00^c^	69.20^b^	96.43^a^	99.60^a^	10.34^d^
Sample size (#)	248	138	215	222	293	25	224	224	249	58
Hub area (km^2^)	19,174	17,812	31,050	24,389	2241	70,280	61,176	11,272	18,901	16,975

The same letter indicates no significant difference at the 5% level. Values in parenthesis are the standard deviations. The dominant cropping system in RL is rice-fallow.

### 3.2 Rice yield and the exploitable yield gap

The mean rice yield in the IL (4.1 t ha^−1^) was almost triple that obtained in the RL (1.4 t ha^−1^) (Table 3). However, exploitable yield gaps between the highest-yielding 10 % of farmers (the top decile) and the mean-yielding farmers (the mean of all farms) were observed in both production systems. The yield gaps were 40 % and 58 % (2.7 t ha^−1^ and 2.0 t ha^−1^) in the IL and the RL, respectively.

**Table 3:** Sample mean, attainable (top decile) mean and gaps in grain yield, net profit and labor productivity in irrigated lowland production systems and irrigated versus rainfed lowland production systems in SSA countries.

The same letter indicates no significant difference at the 5% level.

**Fig. 2:**
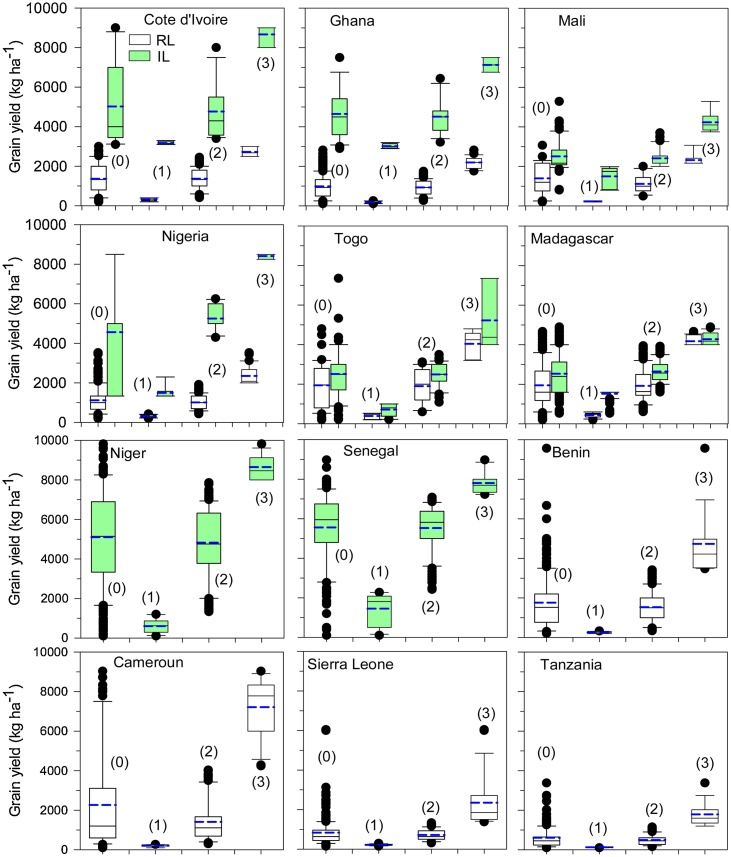
Box-whisker plots of rice yield in irrigated lowland (IL) and in rainfed lowland (RL) production systems: the mean farmer population (0), the bottom 10 % (1), middle 80 % (2), and top 10 % (3) of farmers in 12 countries in SSA. Six countries (Cote d’Ivoire, Ghana, Mali, Nigeria, Togo and Madagascar) had both IL and RF, two countries (Niger and Senegal) had only the IL and four countries (Benin, Cameroon, Sierra Leone, and Tanzania) had only the RL represented in the survey. For the detail survey site name in the respective country, see Table 1, Table 2.

For the IL, the highest rice yield was obtained in Dagana in Senegal (5.5 t ha^−1^), followed by Tillaberi in Niger (5.1 t ha^−1^) and Gagnoa in Cote d’Ivoire (5.0 t ha^−1^), and the lowest yield (2.5 t ha^−1^) was obtained in Kouroumari in Mali, Region Maritimes in Togo and Ankazomiriotra in Madagascar (Fig. 2; Table 2). The exploitable yield gap for the eight countries using the IL ranged between 29–52 % (1.7 to 3.8 t ha^−1^) (Table 3). The highest yield gap in rice was determined for Togo (52 %), followed by Nigeria (46 %), and the lowest were observed in Senegal (29 %) and Ghana (35 %). The highest variability in grain yield was observed in Niger, followed by Senegal, and the lowest variability was observed in Madagascar and Mali (Fig. 2). By AEZ, farmers in the arid zone had the highest yield (5.5 t ha^−1^), and the lowest yield was calculated for the highland zone (2.5 t ha^−1^) (Table 5).

For the RL, the mean rice yield was the highest in Cameroon (2.3 t ha^−1^), and the lowest was (<1 t ha^−1^) in Tanzania, Sierra Leone and Ghana (Fig. 2; Table 4). The exploitable yield gap ranged between 40–69 % (0.9 to 4.9 t ha^−1^) (Table 4). The highest yield gap was calculated for Cameroon (69 %), followed by Tanzania (66 %), and the lowest were for Mali (40 %) and Cote d’Ivoire (50 %). The highest variability in grain yield was observed in Cameroon and the lowest variability was observed in Ghana and Mali (Fig. 2). The highest yield (1.9 t ha^−1^) was calculated in the highland zone, and the lowest was calculated in the subhumid zone (1.1 t ha^−1^) (Table 5).

**Table 4:** Sample mean, attainable (top decile) mean and gaps in grain yield, net profit and labor productivity in rainfed lowland production systems in SSA countries.

The same letter indicates no significant difference at the 5% level.

**Table 5:** Five performance indicators of rice production sustainability across five climatic zones and two production systems.

	Irrigated lowland					Rainfed lowland			
	Arid	Highlands	Humid	Semiarid	Subhumid	Highlands	Humid	Semiarid	Subhumid
Grain yield (kg ha^−1^)									
Population mean	5559^b^	2536^c^	4878^ab^	4698^a^	3240^d^	1949^c^	1505^b^	1398^ab^	1133^a^
Attainable amount	7803	4281	8667	8187	5922	4178	4620	2321	2799
Exploitable gap	2244	1745	3789	3489	2682	2229	3116	923	1666
Gap (%)	29	41	44	43	45	53	67	40	60
Net profit ($ ha^−1^)									
Population mean	796^b^	336^c^	1293^d^	1019^a^	1003^a^	223^b^	337^c^	106^ab^	136^a^
Attainable amount	1275	655	2159	1952	1776	622	1207	396	692
Exploitable gap	479	319	866	933	773	399	871	291	556
Gap (%)	38	49	40	48	44	64	72	73	80
Labor productivity (kg labor day^−1^)									
Population mean	192^c^	22^a^	46^ab^	100^d^	64^b^	29^c^	16^a^	53^d^	24^b^
Attainable amount	190	37	75	188	146	99	40	106	56
Exploitable gap	−3	15	29	88	82	70	24	53	32
Gap (%)	−2	41	39	47	56	71	60	50	57
NUE (kg grain kg^−1^N)									
Population mean	129^a^	–	143^ab^	125^a^	113^b^	–	111^a^	67^a^	44^a^
Attainable amount	170	–	239	176	88	–	188	229	74
Exploitable gap	41	–	97	51	−24	–	77	161	30
Gap (%)	24	–	41	29	−27	–	41	70	41
PUE (kg grain kg^−1^N)									
Population mean	968^a^	–	714^ab^	591^a^	692^b^	–	636^a^	482^a^	242^a^
Attainable amount	1193	–	153	901	838	–	906	1375	338
Exploitable gap	225	–	−561	309	146	–	270	893	96
Gap (%)	19	–	−367	34	17	–	30	65	28

The same letter indicates no significant difference at the 5% level.

NUE and PUE values are compared only in Cote d’Ivoire, Mali, Togo, Niger, Senegal and Benin.

### 3.3 Profit and the exploitable profit gap

Like yield, the mean net profit from the RL ($223 ha^−1^) was significantly lower than that from the IL ($1036 ha^−1^) (Table 3). The profit gaps (derived from the three yield categories) were 39 % in the IL and 72 % in the RL. The values of the exploitable gaps (approximately $600 ha^−1^) were similar in the two production systems. In the RL, 10 % of farmers in the lowest decile had an average negative net profit ($-130 ha^−1^), while this value was $189 ha^−1^ for farmers under IL conditions.

**Fig. 3:**
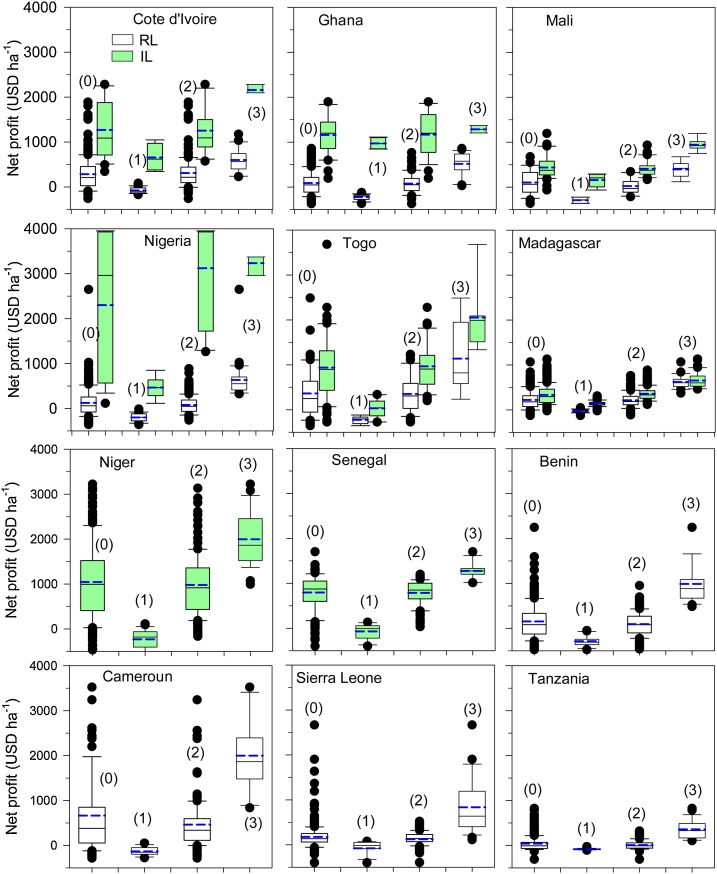
Box-whisker plots of net profits in rainfed lowland (RL) and irrigated lowland (IL) production systems: the mean farmer population (0), the bottom 10 % (1), middle 80 % (2), and top 10 % (3) of farmers in 12 countries in SSA.

For the IL, the highest net profit was obtained in Nigeria ($2301 ha^−1^) and the lowest were obtained in Mali ($440 ha^−1^) and Madagascar ($336 ha^−1^) (Fig. 3; Table 3). The exploitable profit gap ranged between 10–54 % ($128 to 1114 ha^−1^) (Table 3). The highest profit gap was calculated for Togo (54 %), followed by Mali (53 %), and the lowest were calculated for Ghana (10 %) and Nigeria (29 %). The greatest variability in grain profit was observed in Nigeria, followed by Niger, and the lowest variability was observed in Madagascar and Mali (Fig. 3). The highest profits were achieved by farmers in the humid zone ($1293 ha^−1^), and the lowest were achieved in the highland zone ($336 ha^−1^) (Table 5).

For the RL, the highest profits were achieved in Cameroon ($662 ha^−1^) and Togo ($365 ha^−1^), and the lowest profits were achieved in Tanzania ($38 ha^−1^), Ghana ($91 ha^−1^) and Mali ($106 ha^−1^) (Fig. 3; Table 4). Large exploited profit gaps were calculated, and they ranged between 52–89 % ($291 ha^−1^ to $1334 ha^−1^). The highest profit gap in the RL was calculated for Tanzania, and the lowest was calculated for Cote d’Ivoire (Table 4). The greatest variability in profit was observed in Cameroon and Sierra Leone, and the lowest was observed in Madagascar and Mali (Fig. 3). The highest profits ($337 ha^−1^) were obtained in the humid zone, and the lowest profits were obtained in the semiarid zone ($106 ha^−1^) (Table 5).

### 3.4 Labor productivity and the exploitable labor productivity gap

Among the surveyed countries, the average labor productivity was 90 kg grain labor day^−1^ in the IL and only 25 kg grain labor day^−1^ in the RL (Table 3). The low labor productivity in the RL was due to the low grain yields. However, an exploitable gap existed for both production systems. The labor productivity gaps were 38 % and 59 % in the IL and RL, respectively. The exploitable gap in value was higher in the IL system (54 kg grain labor day^−1^) than in the RL (36 kg grain labor day^−1^).

**Fig. 4:**
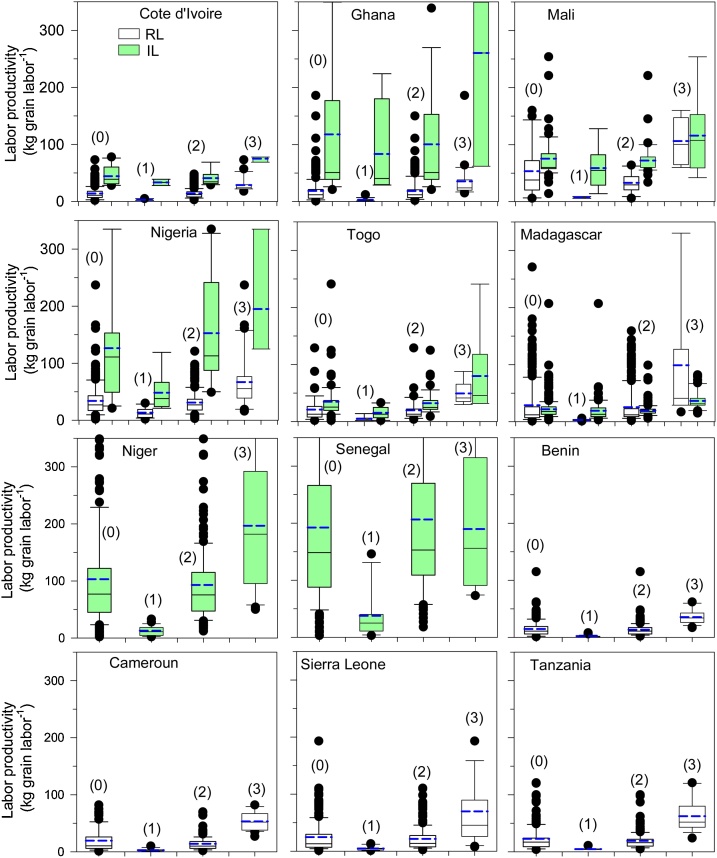
Box-whisker plots of labor productivity in rainfed lowland (RL) and irrigated lowland (IL) production systems: the mean farmer population (0), the bottom 10 % (1), middle 80 % (2), and top 10 % (3) of farmers in 12 countries in SSA.

Under IL conditions, the highest labor productivity was observed in Senegal (192 kg grain labor day^−1^), followed by Nigeria (126 kg grain labor day^−1^) and Ghana (118 kg grain labor day^−1^), and the lowest labor productivity was observed in Madagascar (22 kg grain labor day^−1^) (Fig. 4; Table 3). The exploitable labor productivity gap for the eight countries within IL reached 56 %. The highest labor productivity gap was calculated for Togo (56 %) and Ghana (55 %). The labor productivity gap in Senegal was negative, implying that the farmers producing higher yields had lower labor productivity value than the mean value for the population. The greatest variability in labor productivity was observed in Niger and Senegal, and the lowest variability was observed in Cote d’Ivoire (Fig. 4). Because farmers producing in IL in Senegal had the highest labor productivity, the highest labor productivity (192 kg grain labor day^−1^) was observed in the arid zone; the lowest was observed in the highland zone (22 kg grain labor day^−1^) (Table 5).

For the RL, labor productivity was low in general, ranging from the lowest obtained by farmers in Cote d’Ivoire (14 kg grain labor day^−1^) and Benin (15 kg grain labor day^−1^) to the highest achieved in Mali (53 kg grain labor day^−1^) and Nigeria (34 kg grain labor day^−1^) (Fig. 4; Table 4). Large, exploitable labor productivity gaps existed in the 10 countries with RL, and their values ranged between 47 % (Ghana) and 71 % (Madagascar) (Table 4). The greatest variability in labor productivity was observed in Madagascar and Nigeria, and the lowest variability was observed in Benin and Cameroon (Fig. 4). Among the different AEZ, the highest labor productivity (53 kg grain labor day^−1^) was observed in the semiarid zone, and the lowest (16 kg grain labor day^−1^) was observed in the humid zone (Table 5).

### 3.5 Nitrogen and phosphorus use efficiencies and nutrients mining

The NUE was 146 kg grain kg^−1^ N in IL and 63 kg grain kg^−1^ N in RL (Table 3). The PUE was 754 kg grain kg^−1^ P in IL and 393 kg grain kg^−1^ P in RL. In addition, high NUE and PUE gaps were observed under RL conditions. The NUE gap was only 9% in IL but was as high as 53 % in RL. Approximately 43 % and 20 % of farmers are mining soil N nutrient in the IL and RL production systems, respectively, due to the high values of NUE (>100 kg grain kg^−1^ N) (Table 6). The PUE gap was 46 % in RL (Table 3). The lowest PUE gap calculated in the IL was negative, meaning that the farmers had too high PUE values, which can explain that most farmers are mining soil P nutrient. Approximately 34 % and 61 % of farmers are mining soil P nutrient in RL and IL, respectively, according to their high values of PUE (>400 kg grain kg^−1^ P) (Table 6).

**Table 6:** Percentage of farmers in different categories of nitrogen (NUE) and phosphorus use efficiency (PUE) in irrigated and rainfed lowland production systems in SSA countries.

	Overall		Irrigated					Rainfed lowland			
	Irrigated lowland	Rainfed lowland	Cote d’Ivoire (Gagnoa)	Mali (Kouroumari)	Niger (Tillaberi)	Senegal (Dagana)	Togo (Maritime)	Benin (Glazoue)	Cote d’Ivoire (Man)	Mali (Sikasso)	Togo (Plateaux)
Nitrogen use efficiency (NUE, kg grain kg-1 elemental N)											
Too low (wasteful application, < 30)	13.78	43.54	12.50	2.22	16.30	11.86	15.09	58.94	14.72	47.37	71.15
Desirable range (30–100)	43.70	36.28	31.25	31.11	46.38	41.53	49.06	29.47	47.85	36.84	26.92
Too high (soil mining, > 100)	42.52	20.18	56.25	66.67	37.32	46.61	35.85	11.59	37.42	15.79	1.92
Phosphorus use efficiency (PUE, kg grain kg-1 elemental P)											
Too low (wasteful application, <100)	4.79	25.94	0.00	0.00	6.51	2.00	2.17	37.31	3.82	14.29	41.18
Desirable range (100–400)	34.26	39.80	27.27	13.79	39.85	20.00	32.61	44.78	30.53	42.86	43.14
Too high (soil mining, > 400)	60.96	34.26	72.73	86.21	53.64	78.00	65.22	17.91	65.65	42.86	15.69

For NUE, Too low (wasteful application, <30); Desirable range (30-100); Too high (soil mining, >100).

For PUE, Too low (wasteful application, <100); Desirable range (100-400); Too high (soil mining, >400).

**Fig. 5:**
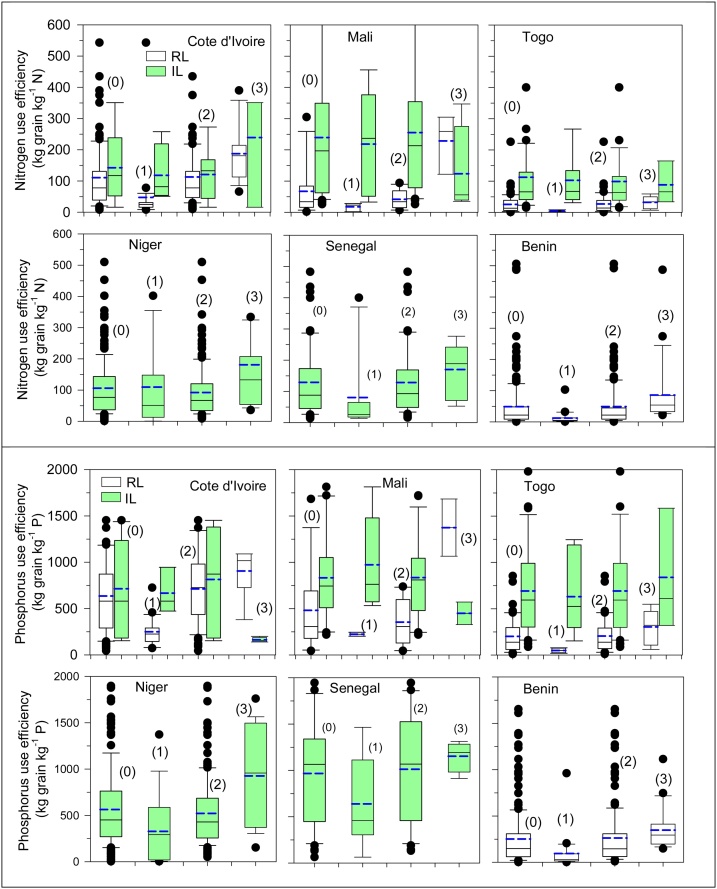
Box-whisker plots of nitrogen (upper two rows) and phosphorus (lower two rows) use under rainfed lowland (RL) and irrigated lowland (IL) production systems in 6 countries in SSA.

Among the five countries for which the NUE under IL conditions was calculated, Mali had the highest NUE (239 kg grain kg^−1^ N), and Niger had the lowest (106 kg grain kg^−1^ N) (Fig. 5; Table 3). The exploitable NUE gap reached 41 % in Niger and Cote d’Ivoire. The lowest NUE gap was negative, implying that the farmers in the top 10 % by yield had below-average NUE and majority of farmers apply too low amount of those nutrients from inorganic fertilizers. Indeed, most farmers are mining soil nutrients. Only 44 % of farmers were within an acceptable NUE range (30–100); 42 % had high values of NUE, indicating soil nutrients mining conditions, and the remaining farmers (14 %) had low NUE, indicating wasteful nutrient management practices (Table 6). The highest percentages of farmers with high NUE values (mining soil nutrients) were observed in Mali (66 %) and Cote d’Ivoire (56 %). The greatest variability in NUE was in observed in Mali and Niger, and the lowest variability was observed in Cote d’Ivoire (Fig. 5). Among the different AEZ, no zone had the average in the optimal NUE range (30–100) and the highest NUE (143 kg grain kg^−1^ N) was found in the humid zone (Table 5). The average PUE was above the optimal PUE range in the five countries with the highest PUE (mining soil nutrients) was in Senegal (968 kg grain kg^−1^ P), and the lowest was in Niger (564 kg grain kg^−1^ P) (Fig. 5; Table 3).

For the rainfed lowland production system, the highest NUE and PUE were achieved in Cote d’Ivoire (111 kg grain kg^−1^ N and 636 kg grain kg^−1^ P, respectively) (Fig. 5; Table 4), indicating both N and P mining are happening in Cote d’Ivoire as these values are above the upper limits. Similarly, the lowest NUE and PUE were observed in Togo (26 kg grain kg^−1^ N and 202 kg grain kg^−1^ P, respectively), indicating overapplication by farmers (71 % for NUE and 41 % for PUE). The farmers in the top 10 % by yield in Benin and Togo are in the optimal range of both NUE and PUE. Approximately 44 % had low values of NUE (waste of N fertilizers) with the highest percentage in Benin and Togo (Table 6).

### 3.6 Trade-offs among indicators in countries and production systems

**Fig. 6:**
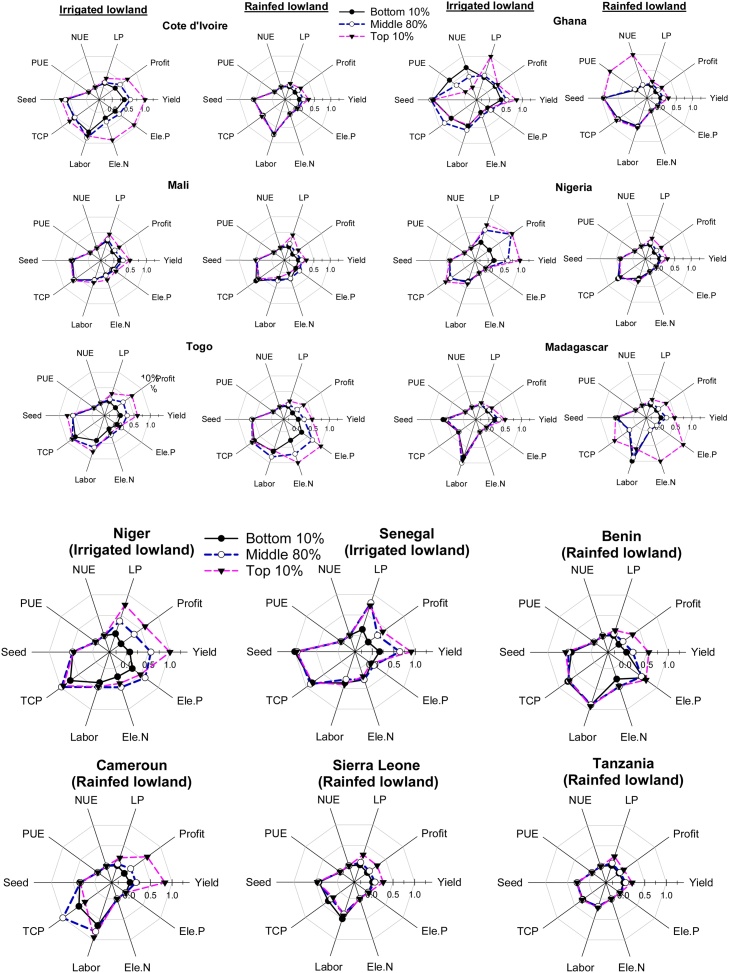
Trade-offs among different indicators and inputs for farmers in three yield gap categories applying under irrigated and rainfed lowlands in 12 countries in SSA. Indicators: yield = grain yield, profit = net profit, LP = labor productivity, NUE = nitrogen use efficiency, and PUE = phosphorus use efficiency. Input values: seed = seeding rate (kg ha^−1^), TCP = total cost of production ($ ha^−1^), labor = no. of labor days ha^−1^, Ele. N = elemental N kg ha^−1^, and Ele. P = elemental P kg ha^−1^. The values are the averages for the bottom 10 %, top 10 % and middle 80 %. NUE and PUE values are compared only for Cote d’Ivoire, Mali, Togo, Niger, Senegal and Benin. Cameroon, Madagascar, Sierra Leone, and Tanzania were excluded from this figure because they had very high or low NUE and PUE values.

**Fig. 7:**
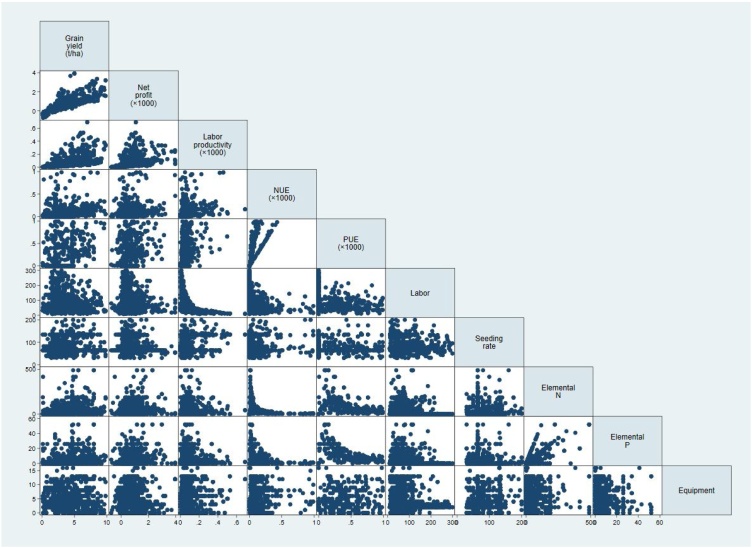
Scatter plot of the correlation between performance indicators and production inputs in the irrigated lowland production systems.

**Fig. 8:**
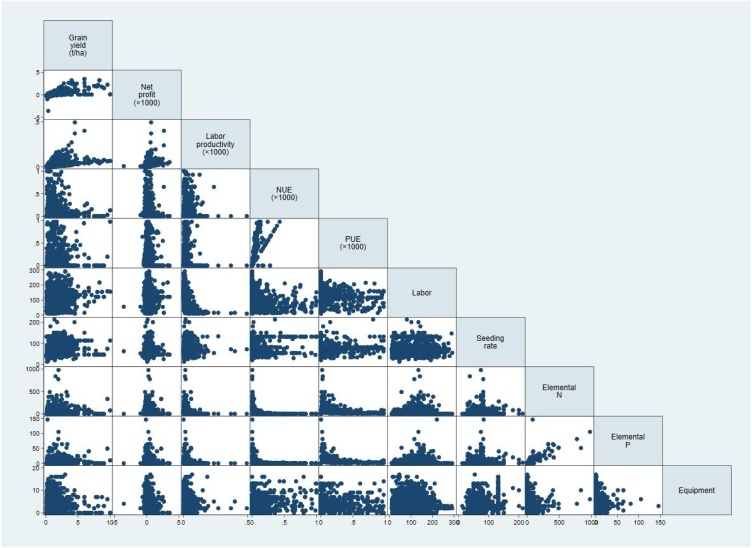
Scatter plot of the correlation between performance indicators and production inputs in the rainfed lowland production systems.

The analyses highlighted the trade-offs among the five PIs and production inputs based on the three farmer categories (Fig. 6). In the IL production system, clear differences in the PIs and production inputs among the three yield categories were observed in Cote d’Ivoire, Mali, Togo, Nigeria, Niger and Senegal. In addition to producing higher yields, the top 10 % yielding farmers had higher profits and labor productivity than the farmers in the two other yield categories (bottom 10 % and middle 80 %) in the following six countries: Cote d’Ivoire, Mali, Togo, Nigeria, Niger and Senegal. The correlation analysis also confirmed that in both irrigated and rainfed production systems, there were strong positive correlations between yield, net profit and labor productivity (Table 7, Table 8; Fig. 7, Fig. 8). The quantities of N and P fertilizers were also positively correlated with three PIs (yield, profit and labor productivity) in IL and RL production systems (Table 7, Table 8). However, there was no significant correlation between the two efficiencies (NUE and PUE) and two PIs (yield and labor productivity). In IL, the quantity of labor use was negatively correlated with the use of equipment and herbicide while the labor productivity was positively correlated with the use of equipment and herbicide (Table 7). The top 10 % of farmers in Cote d’Ivoire, Mali, Togo and Niger used higher quantities of N and P fertilizers than the other farmer categories, but this was not the case in Nigeria and Senegal. The top 10 % of farmers in Cote d’Ivoire, Togo and Niger also used more labor than the rest of the farmers, while the top 10 % in Cote d’Ivoire and Togo used higher quantities of seeds than the rest of the farmers.

**Table 7:** Correlation coefficients between performance indicators and production inputs in the irrigated lowland production system.

Variables	Grain yield	Net profit	Labor productivity	NUE	PUE	Labor	Seeding rate
Net profit	0.776***						
Labor productivity	0.603***	0.429***					
NUE	0.249***	0.264***	0.346***				
PUE	0.450***	0.443***	0.484***	0.757***			
Labor	−0.237***	−0.197***	−0.585***	−0.363***	−0.489***		
Seeding rate	0.173***	−0.001	0.300***	0.141***	−0.069*	−0.185***	
Elemental N	0.351***	0.282***	0.173***	−0.105***	0.266***	−0.190***	0.010
Elemental P	0.318***	0.319***	0.093***	−0.068**	0.253***	−0.153***	−0.137***
Equipment use	0.148***	0.141***	0.201***	0.227***	0.383***	−0.282***	0.190***
Herbicide	0.167***	0.215***	0.180***	0.266***	0.610***	−0.335***	0.077**

*p < 0.1, **p < 0.05, ***p < 0.01.

**Table 8:** Correlation coefficients between performance indicators and production inputs in the rainfed lowland production systems.

Variables	Grain yield	Net profit	Labor productivity	NUE	PUE	Labor	Seeding rate
Net profit	0.711***						
Labor productivity	0.427***	0.376***					
NUE	−0.035	−0.025	0.086***				
PUE	0.100***	0.040	−0.043	0.749***			
Labor	0.273***	0.118***	−0.389***	−0.154***	0.088***		
Seeding rate	0.102***	−0.013	−0.020	0.312***	0.235***	0.201***	
Elemental N	0.159***	0.046**	−0.046**	−0.107***	0.081***	0.207***	0.048**
Elemental P	0.150***	0.042*	−0.056**	−0.105***	0.060**	0.236***	0.054**
Equipment use	−0.079***	−0.069***	−0.008	0.236***	0.183***	−0.096***	0.309***
Herbicide	−0.027	−0.064***	−0.045**	0.472***	0.570***	0.038*	0.321***

*p < 0.1, **p < 0.05, ***p < 0.01.

In RL, the top 10 % of farmers in terms of yield also had higher profits and labor productivity than farmers in the other yield categories (bottom 10 % and middle 80 %) in all ten countries (Cote d’Ivoire, Ghana, Madagascar, Mali, Nigeria, Togo, Benin, Cameroon, Sierra Leone, and Tanzania). The top 10 % of farmers in Benin, Madagascar and Togo used higher quantities of N and P fertilizers than the rest of the farmers. They also used a higher quantity of labor in Cameroon but less labor in Madagascar. This implies that low-performing farmers in Madagascar should use more N and P fertilizers and could reduce labor inputs; those in Togo and Benin should use more N and P fertilizers; and those in Cameroon should increase labor inputs.

## 4 Discussion

Estimating economic and environmental performance indicators of rice production is a first step for identifying intervention areas across countries, production systems and AEZ to improve productivity and profitability, reduce drudgery, increase sustainability of rice production (Devkota et al., 2019). The assessment of rice production systems in 12 SSA countries showed a large variation in rice yield across countries and between production systems. The mean yield varied widely between 2.5 to 5.6 t ha^−1^ and 0.6 to 2.3 t ha^−1^ in IL and RL, respectively. In addition, rice yields are critically low, especially in RL, where farmers produced, on average, 1.4 t ha^−1^. Although the average yield (4.1 t ha^−1^) is higher in IL, farmers produced, on average, only 2.5 t ha^−1^ in IL in Madagascar, Mali and Togo. Result of lower yield of RL than IL confirm previous studies (Niang et al., 2017; Tanaka et al., 2017; Senthilkumar et al., 2020). While comparing the rice yield in SSA countries with the intensively managed lowland rice yield in six East Asia countries (Devkota et al., 2019), yields in both IL and RL are lower than that of many sites in Asia except for Bago site in Myanmar. In SSA countries, lower yields were associated with no or lower N and P fertilizer applications in this study. This finding is supported by studies on nutrition omission trials or fertilizer response trials in SSA (Saito et al., 2019; Tsujimoto et al., 2019; Niang et al., 2017). Saito et al. (2019) showed positive response of rice yield to N and P fertilizer, and rice yields without N, P, and K were only 68, 84, and 89 % of yields of the NPK treatment. Nevertheless, a high proportion of farmers do not apply N and P fertilizers or apply only very low quantities. On average, 61 % of farmers in RL and 44 % in IL do not apply N or apply <2 kg N ha^−1^ in rice, while up to 74 % of farmers in RL and 55 % in IL do not apply P or apply <1 kg P ha^−1^ in rice. The cause of low level of fertilizer application is not known in this study, but may be related to combination of the following. First, low level of fertilizer application may be due to high prices coupled with farmers’ financial constraints. When farmers need to buy fertilizers at the beginning of rice growing season, they might have financial liquidity constraints and limited access to financial services or credits. In many SSA countries, governments do not provide financial assistance to farmers and where it is available, it is mainly for cash crops such as coffee, cocoa, cotton, etc. The financial constraint assumption was also raised by Wortmann et al. (2019) in their book chapter that analyzed smallholder farmers’ fertilizer use issues in Africa. Secondly, farmers access and timely availability of fertilizers are usually an issue. Even in countries where subsidies exist, due to poor infrastructure and supply chains, fertilizer inputs may not be available on time. Thirdly, especially for rainfed lowlands, farmers experience high levels of uncertainty about biophysical factors (erratic rainfall, insufficient water in the field, etc.), which are increasing with the negative effect of climate change, leading to the low use of inputs (e.g. fertilizer) (Niang et al., 2018; Arouna et al., 2021b). As soil texture is largely variable at short distance in rainfed lowlands, there is a need for field-specific recommendations that consider soil texture and the spatial-temporal dynamics of water availability to reduce risk and uncertainty about biophysical factors and increase the use of fertilizers (Niang et al., 2018). For drought-prone conditions, water conservation measures, such as bunding, mulching, land-leveling, and no-tillage should also be considered for enhancing soil moisture and improving yield response to fertilizer. In addition, if reliable weather forecasting becomes available, it will help farmers to take timely decision to reduce the risk and uncertainty related to climatic factors. Although the average amount of fertilizer used as determined in this study is lower than the crop required (considering efficiencies), high variability was observed among farmers and production systems. This explains the large yield gaps among different countries and production systems. Relative yield gap of 29–69% and absolute gap of 2.7 t ha^−1^ in IL and 2.0 t ha^−1^ in RL in this study are similar to those from recent field surveys in SSA (Tanaka et al., 2017; Dossou-Yovo et al., 2020) but higher than in Asian countries where the yield gap ranged from 24 to 42 % (Devkota et al., 2019). These demonstrate the high heterogeneity among farmers and suggest that, with moderate changes in the production practices (integrated good agronomic practices), farmers can improve their yields (by following the practices of their peers) in similar socioeconomic and biophysical conditions.

There was a profit gap of 10–89%, and labor productivity gap up to 71 % between the 10 % highest-yielding and the mean-yielding farms. These gaps are generally higher than those computed for IL in six East Asian countries (Devkota et al., 2019). IL had significantly higher performance especially for yield, profit and labor productivity than RL. However, the average cultivated area per farmer under IL is lower than that under RL. This finding indicates the need to increase the cultivated area per household in IL and access to irrigation water in RL to increase rice production in SSA countries. To improve PIs of RL rice production, low-cost land and water management practices, such as the “smart-valley approach” (a participatory water and land management in inland valley landscapes with field leveling and bundling), could be introduced to improve the accessibility to water in lowland and reduce risk for crop failure, leading to more use of fertilizers (Arouna and Akpa, 2019; Rodenburg et al., 2014).

Yield, profit, and labor productivity were positively correlated in both IL and RL. However, correlation coefficients are slightly different between IL and RL. Relationships between labor productivity and the other two indicators were weaker than relationships between yield and profit in both IL and RL. Farmers with high labor productivity may not always be high-yielding farmers. This suggests that it is important to evaluate key performance indicators and evaluate potential trade-off among them for identifying intervention areas for sustainable rice cultivation. It is noted that there were high labor use and large variations in production cost among farmers in this study. Causes behind the high labor use and lower labor productivity can be explained by low adoption of labor-saving technologies such as equipment (for instance mechanical weeders) and herbicide. This confirms the findings of Rodenburg et al. (2019). Indeed, labor use and labor productivity are significantly correlated with the use of equipment and herbicide in the IL. Although we do not have data on a list of all machineries used by farmers, it is known that, in countries such as Senegal, Niger and Nigeria, production activities in IL are mechanized. In this study, these countries have low labor inputs and higher labor productivity (more than 100 kg grain labor day^−1^). Recent studies in Asian countries showed that farmers were more labor efficient due to a high level of adoption of labor-saving technologies (Bordey et al., 2016; Devkota et al., 2019). In addition, there is an increasing labor scarcity in rural areas in SSA due to urban migration. These indicate that there is a scope for reducing labor use and increasing labor productivity through adoption of labor-saving technologies.

Among those applying fertilizer, only 34–44% of farmers belonged to desirable ranges in NUE and PUE, and a significant number of farmers was in the lower or higher thresholds of NUE and PUE. The higher values indicate mining soil nutrients is occurring (Devkota et al., 2019; EU Nitrogen Expert Panel, 2015), whereas lower values indicate that fertilizers are not effective due to some reasons, including poor crop, nutrient, water, and weed managements. The high values of NUE in the 12 study countries are like what was observed in Myanmar, where approximately 92 % of farmers surveyed were engaged in mining soil nutrients (Devkota et al., 2019). For countries having low NUE and PUE with higher N and P application rates, reasons for lower values could be attributed to poor crop, nutrient, water, and weed managements by farmers, which thus calls for dissemination of nutrient management practices together with integrated crop management practices (Saito et al., 2015; Tanaka et al., 2015; Arouna et al., 2021b).

The trade-off analysis reveals country- and production system- intervention areas for improving sustainability performance indicators. For example, larges difference in yield and N and P fertilizer application rates between the 10 % highest-yielding farms and the mean-yielding farms in IL indicate that higher quantities of N and P fertilizers, labor and seeds may help to close yield gaps in Cote d’Ivoire. To improve yields, low performing farmers in yield should increase N and P fertilizers in IL in Niger. In RL, low-performing farmers in Madagascar should increase the quantities of N and P fertilizers applied and decrease labor use, while those in Cameroon should employ more labor. The difference between Cameroon and Madagascar is related to availability and associated cost of labor. In Madagascar, because of labor abundance especially the family labor, low-performing farmers are using too labor, which is reducing the efficiency. In contrast, because of high labor cost, low-performing farmers in Cameroon were using less labor than the top 10 % yielding farmers. In addition, the difference between Madagascar and Cameroon is also due to the actual level of labor input in each country. Indeed, the average labor input is higher in Madagascar (132 labor day ha^−1^) than in Cameroon (122 labor day ha^−1^). It is worth mentioning that although the above discussion focuses on increasing the quantities of N and P fertilizers as short-term solutions, alternative sources to N and P fertilizers such as locally available organic inputs or crop rotation systems with legumes can be also considered for long-term sustainability of rice-based systems.

Although the analysis in this paper gave an in-depth view of the sustainability of the rice production system in SSA, there are some limitations in this study. First, the data used for analysis was for one growing season in 2013–2014. In the future study, data from several years are needed to analyze the temporal changes in the PIs in rice production in SSA. Second, data collection using interview especially for key parameters such as field size and yield, as it is done in this study, may reduce the precision of the estimation of the PIs. If resources permit, measuring tape or map calculation for field size and crop-cut for yield should be preferred (Saito et al., 2021). Lastly, future research could also increase the sample size per site to improve the robustness of the parameter estimates for the top 10 % of farmers.

## 5 Conclusions

Grain yields of the smallholder farmers in irrigated and rainfed lowlands were low in the 12 surveyed countries. The low yields are explained by low levels of nutrient input use. In addition, low nutrient input use resulted in extremely higher NUE and PUE, indicating mining soil nutrients. Dissemination and adoption of integrated crop management practices including nutrient management practices may help improving rice productivity, profit, and nutrient use efficiency. The existence of large yield gaps between the top decile of farmers and the farmer population mean shows that context-based innovations can be developed by following the management practices of high-yielding farmers to improve input management and the sustainability of rice production. Such an approach will lead to innovations that are more adapted to farmers’ socioeconomic and biophysical conditions. The irrigation production system performed better than the rainfed lowland production system. This confirms the importance of water management, especially low-cost approaches such as ‘smart valleys approach’, for reducing risk for drought and flooding, and increasing rice production in SSA. Large profit gaps were also noted in rice production and were due mainly to input costs, especially labor costs. Labor productivity was generally low in rice production in SSA, and it can be improved through introduction of labor-saving technologies.

## Authors statement

**Aminou Arouna:** Conceptualization, Methodology, Writing - Review & Editing; **Krishna Prasad Devkota:** Methodology, Writing - Review & Editing, Formal analysis; **Wilfried Gnipabo Yergo:** Data Curation, Formal analysis; **Kazuki Saito:** Supervision, Writing - Review & Editing; **Benedicta Nsiah Frimpong:** Investigation, Visualization, **Patrice Ygue Adegbola:** Investigation, Writing - Review & Editing, **Ernest Meougbe Depieu:** Investigation, Writing - Original Draft; **Dorothy Malaa Kenyi:** Visualization, Formal analysis; **Germaine Ibro:** Data Curation, Writing - Original Draft; **Amadou Abdoulaye Fall:** Investigation, Visualization; **Sani Usman:** Data Curation.

## Declaration of Competing Interest

The authors report no declarations of interest.
